# Abdominal Obesity and Insulin Resistance in People Exposed to Moderate-to-High Levels of Dioxin

**DOI:** 10.1371/journal.pone.0145818

**Published:** 2016-01-11

**Authors:** Jung-Wei Chang, Hsiu-Ling Chen, Huey-Jen Su, Ching-Chang Lee

**Affiliations:** 1 Research Center for Environmental Trace Toxic Substances, National Cheng Kung University, Tainan, Taiwan; 2 Department of Industrial Safety and Health, Hung Kuang University, Taichung, Taiwan; 3 Department of Environmental and Occupational Health, College of Medicine, National Cheng Kung University, Tainan, Taiwan; University of Texas Health Science Center at San Antonio, UNITED STATES

## Abstract

Obesity, a risk factor for developing metabolic complications, is a major public health problem. Abdominal obesity is strongly accompanied by a cluster of metabolic abnormalities characterized by insulin resistance. The link between persistent organic pollutants (POPs) and insulin resistance has been investigated in animal and epidemiological studies. We aimed to examine whether insulin resistance is greater in people with abdominal obesity (AO) and concomitant exposure to serum dioxins (PCDD/Fs). We conducted a cross-sectional descriptive study of 2876 participants living near a PCDD/Fs contaminated area. Seventeen 2,3,7,8-substituted PCDD/Fs congeners were measured, and then the associations between the main predictor variable, serum TEQ_DF-1998_, abdominal obesity (AO), dependent variables, and insulin resistance were examined. Twelve of the 17 congeners, widely distributed among PCDDs, and PCDFs, had trends for associations with abdominal adiposity. In men, the highest quintiles of 1,2,3,7,8-PeCDF; 1,2,3,7,8-PeCDD; 2,3,7,8-TCDD; 2,3,7,8-TCDF; and 2,3,4,7,8-PeCDF had the top five adjusted odds ratios (AORs) + 95% confidence intervals (CIs):[4.2; 2.7–6.4], [3.6; 2.3–5.7], [3.2; 2.1–5.0], [3.0; 2.0–4.5], and [2.9; 1.9–4.7], respectively. In women, the highest quintiles of 1,2,3,4,7,8,9-HpCDF; 1,2,3,6,7,8-HxCDF; and 1,2,3,4,6,7,8-HpCDF had the top three AORs + 95% CIs:[3.0; 1.9–4.7], [2.0; 1.3–3.1], and [1.9; 1.3–2.9], respectively. After confounding factors had been adjusted for, men, but not women, with higher serum TEQ_DF-1998_ levels or abdominal obesity had a significantly (P_trend_ < 0.001) greater risk for abnormal insulin resistance. The groups with the highest joint serum TEQ_DF-1998_ and abdominal obesity levels were associated with elevated insulin resistance at 5.0 times the odds of the groups with the lowest joint levels (AOR 5.23; 95% CI: 3.53–7.77). We hypothesize that serum TEQ_DF-1998_ and abdominal obesity affect the association with insulin resistance in general populations.

## Introduction

Obesity is the leading cause of numerous metabolic complications (diabetes, cardiovascular diseases, etc.), so it naturally occupies a spot as one of the most pressing public health concerns. The result of an interplay between genetic and environmental factors contribute to this disorder. They include: decline in physical activity, and consuming too many calories. In addition, the food contaminants are accountable for the obesity epidemic and the resulting metabolic responses. Abdominal obesity activates insulin resistance (IR), which will cause the metabolic adaptations that comprise metabolic syndrome [1, 2]. However, the association between abdominal obesity and characteristics of the metabolic syndrome, evidently changes with gender [3, 4], and with different levels of obesity [5, 6]. Obesogens are frequently endocrine disruptors and belong to several chemical families. Several studies report that persistent organic pollutants (POPs), which are either dioxin-like (DL) or non-DL, affect dioxin receptors (AhRs; aryl hydrocarbon receptors) much like dioxins do. Rodent models indicate that DL chemicals may be obesogens. Exposure to TCDD (2,3,7,8-tetrachlorodibenzo-*p*-dioxin) (100 μg/kg of body weight [BW]) once every 2 weeks for 8 weeks, increased the body weights of adult mice more than 40% higher than those of controls [7]. This body weight change was only observed in high-fat diet-fed mice. In a one-month study, the body weights of mouse pups on postnatal days 16–20 increased after exposure to the polychlorinated biphenyl (PCB) mixture (Aroclor 1254) [8]. Further, exposing adult mice to PCB-77 led to an AhR-dependent increase in body mass [9] and increased total lipid content in fatty livers in a cardiovascular disease mouse model [10].

Until recently, body mass index (BMI) has been the tool for classifying obesity, but recently it has been criticized because it does not explain the alterations in body fat distribution and abdominal fat mass, which can fluctuate considerably within a restricted range of BMI. Excess visceral fat is at an increased risk of obesity-related morbidity compared to overall adiposity. Thus, measuring waist circumference (WC) and the waist-hip ratio have been considered a better index than BMI when exploring the risks associated with obesity. WC is now considered the best simple measure of both visceral fat mass and total fat [11]. The influence of body fat on insulin action is important, and the association between obesity and insulin resistance plus type 2 diabetes mellitus (DM) has long been recognized [12]. The relationship between insulin resistance and obesity, and abdominal obesity in notable, is well established [13], and it is believed by some that abdominal obesity is a major contributor to developing MetS [14]. In addition, the link between POPs and insulin resistance has also been investigated in animal and epidemiological studies [15–17]. We previously [17–19] reported on a group of residents from a PCDD/F-contaminated area with several chronic diseases: insulin resistance, MetS, and hyperuricemia. We hypothesized that the residents had a high risk of abdominal obesity and insulin resistance because they were exposed to higher than normal levels of PCDD/Fs. Therefore, the aim of this work was to study the association between PCDD/Fs exposure and abdominal obesity, and to examine whether insulin resistance is greater when abdominal obesity and serum PCDD/F levels are high.

## Materials and Methods

### Participants and procedures

We conducted a cross-sectional descriptive study from July 2005 through May 2010 in a district health center near a PCDD/Fs contaminated area [17–19]. From the previous studies, the residents had a high risk of PCDD/Fs exposure from eating contaminated seafood. The primary recruitment criterion, in addition to age and an agreement to provide the amount of blood required for the study, was that the participant had to reside in the exposure area. The 3128 participants consisted of approximately 85% of all invited residents over 17 years old in the exposure area. Reasons for participation and non-participation are not associated with their PCDD/Fs exposure [17–19]. Of the initial 3128 study participants, we excluded 217 who did not provide their waist or hip circumference. Of the initial 2911 study participants, we excluded 35 who had at least one of following diseases: liver cirrhosis (n = 9, 0.3%), rheumatoid arthritis (n = 24, 0.8%), and systemic lupus erythematosus (SLE)(n = 2, 0.1%). There was no acute inflammation case in subject recruited in this study. Finally, 2876 participants met the inclusion criteria. Details of the study’s protocol and all testing procedures are available in our previous studies [17–19]. This survey was approved by the Institutional Review Board of National Cheng Kung University Hospital; signed informed consent was obtained from all participants.

Anthropometrical measurements including height, weight, body fat, and waist and hip circumference were recorded using standard procedures. The waist circumference measured at the point midpoint between the lower rib margin and the iliac crest and hip circumference at the widest circumference of the buttocks. A waist/hip ratio (WHR) of > 0.8 in women and 0.9 in men suggests abdominal obesity.

Participants were asked to fast the night before 80-mL samples of venous blood were drawn. Information obtained from the questionnaire included personal characteristics (age, gender, medical history of major systemic diseases), and current lifestyle habits (alcohol consumption, smoking, eating habits, etc.). The body fat percentage and body weight was analyzed by body fat analyzer (HBF-352; Omron, Tokyo, Japan).

### Laboratory procedures

We collected blood samples using vacuum tubes without anticoagulants. And then the samples were analyzed by isotope dilution high-resolution gas chromatography/high-resolution mass spectrometry (HRGC/HRMS), as previously described [17–19]. All PCDD/Fs were adjusted to the lipid content analyzed from the corresponding samples. Serum total cholesterol, high-density lipoprotein (HDL) cholesterol, and triglycerides were determined in the central laboratory of National Cheng Kung University Hospital using an auto analyzer (747E; Hitachi Koki Co., Tokyo, Japan).

### Data processing and statistical analysis

PCDD/F concentrations are expressed in picograms (pg = 10^−12^ gram) WHO_1998_-TEQ_DF_/g lipid. The ln-transformed serum TEQ_DF-1998_ improved the normality (checked using Q-Q plots) and homogeneity of variance, and the statistical analysis was done using the ln-transformed data. All values below the limit of detection were treated as half of this limit. JMP 5.0 (SAS Institute, Cary, NC) was used for all statistical analyses. Unless indicated otherwise, data are mean ± standard deviation (SD). Categorical variables were compared using χ^2^ or Fisher’s Exact tests. Continuous variables were compared using the Wilcoxon Rank-Sum test. Correlations between anthropometry measurement, biochemistry examination, and serum TEQ_DF-1998_ were tested using linear regression and expressed by Pearson’s correlation coefficient. All statistical tests were two-tailed; significance was set at *P* < 0.05 (two-tailed). The association between serum TEQ_DF-1998_ and abnormal insulin resistance (HOMA-IR) was assessed by multiple logistic regression. And the potential confounders included in the models were age (< 40, 40–60, > 60 years), gender, cigarette smoking history (ever/never), alcohol drinking history (yes/no), physical activity, abnormal levels of triglycerides and cholesterol. We examined the associations of HOMA-IR with serum TEQ_DF-1998_ using receiver operating characteristic (ROC) analyses in conjunction with Youden’s index [20]. The index is defined for all points of an ROC curve, and the maximum value of the index may be used as a criterion for selecting the optimal cutoff point when a diagnostic test gives a numeric rather than a dichotomous result. The participants were then split into 4 groups according to the cutoff level of serum TEQ_DF-1998_ and the WHR. Adjusted odds ratios (AORs) were calculated using the lower serum TEQ_DF-1998_ and no abdominal obesity as the reference group. Potential interaction of serum TEQ_DF-1998_ and abdominal obesity was evaluated by adding an interaction term of “abdominal obesity × TEQ_DF-1998_” into the multiple logistic regression model. Significance for the interaction term was set at *P* < 0.01.

## Results

We divided the 2876 participants (1466 men and 1410 women) whose data were available into those with abdominal obesity (AO^Pos^) and without abdominal obesity (AO^Neg^) based on Taiwan DOH criteria for waist-to-hip ratio (WHR) (Table 1). The average mean age of the men (847 AO^Neg^; 619 AO^Pos^) was 45.9 years (AO^Neg^: 40.6 ± 15.9; AO^Pos^: 53.1 ± 16.4; *P <* 0.001) and of the women (666 AO^Neg^; 744 AO^Pos^) was 46.6 years (AO^Neg^: 38.0 ± 13.4; AO^Pos^: 54.3 ± 17.1; *P <* 0.001) (Table 1).

**Table 1:** Demographic characteristics in participants with abdominal obesity (AO^Pos^) and without (AO^Neg^). [Mean (SD) or Number (%)].

Characteristic	Men			Women		
	AO^Neg^	AO^Pos^	*P*	AO^Neg^	AO^Pos^	*P*
	(n = 847)	(n = 619)		(n = 666)	(n = 744)	
Age (years)	40.6 (15.9)	53.1 (16.4)	< 0.001	38.0 (13.4)	54.3 (17.1)	< 0.001
Body mass index (kg/m^2^)	23.3 (3.4)	27.1 (4.0)	< 0.001	21.9 (3.4)	25.9 (4.0)	< 0.001
Body fat (%)	21.6 (5.3)	27.8 (4.3)	< 0.001	28.9 (4.8)	35.4 (4.6)	< 0.001
Waist circumference (cm)	80.9 (7.9)	96.0 (8.5)	< 0.001	70.6 (7.0)	86.4 (9.2)	< 0.001
Hip circumference (cm)	96.4 (6.8)	100.7 (7.9)	< 0.001	94.2 (7.5)	99.2 (9.0)	< 0.001
Waist-hip ratio (cm/cm)	0.84 (0.05)	0.95 (0.06)	< 0.001	0.75 (0.04)	0.87 (0.06)	< 0.001
Smoking (%)	440 (52.0%)	392 (63.3%)	< 0.001	31 (4.7%)	31 (4.2%)	0.656
Drinking (%)	188 (22.2%)	215 (34.7%)	< 0.001	17 (2.6%)	18 (2.4%)	>0.873
Systolic BP (mm Hg)	120.4 (17.8)	131.4 (20.5)	< 0.001	111.7 (17.4)	132.4 (26.1)	< 0.001
Diastolic BP (mm Hg)	74.0 (10.8)	79.7 (11.6)	< 0.001	68.2 (9.9)	75.8 (12.2)	< 0.001
Serum TEQ_DF-1998_	21.9 (38.3)	30.3 (33.5)	< 0.001	23.6 (29.4)	44.8 (57.9)	< 0.001

Abbreviations: AO = abdominally obese; BP = blood pressure; TEQ_DF-1998_ = pg WHO_1998_-TEQ_DF_/g lipid, toxic equivalency of PCDDs (D) and PCDFs (F); 1998 = World Health Organization 1998 toxic equivalency factors.

*p*: indicates whether demographic characteristics and serum PCDD/Fs differ by AO status (Wilcoxon Rank-Sum test for continuous variables and χ^2^ test for categorical variables).

AO^Pos^ men had significantly larger anthropometric values for BMI, body fat, waist and hip circumferences, and blood pressure (all *P <* 0.001). In addition, serum TEQ_DF-1998_ was significantly lower in AO^Neg^ than in AO^Pos^ men (AO^Neg^: 21.9 ± 38.3; AO^Pos^: 30.3 ± 33.5 pg WHO_1998_-TEQ_DF_/g lipid; *P* < 0.001). AO^Pos^ women had significantly larger anthropometric values for BMI, body fat, waist and hip circumference, and blood pressure (all *P <* 0.001). In addition, the serum TEQ_DF-1998_ was significantly lower in AO^Neg^ than in AO^Pos^ women (AO^Neg^: 23.6 ± 29.4; AO^Pos^: 44.8 ± 57.9 pg WHO_1998_-TEQ_DF_/g lipid; *P* < 0.001).

AO^Pos^ men had higher cholesterol (AO^Pos^: 201.4 ± 41.7; AO^Neg^: 186.5 ± 40.2 mg/dL; *P <* 0.001) and triglycerides (AO^Pos^: 195.4 ± 255.8; AO^Neg^: 127.5 ± 123.7 mg/dL; *P <* 0.001). Moreover, AO^Pos^ women had higher cholesterol (AO^Pos^: 203.7 ± 44.0; AO^Neg^: 187.8 ± 40.5 mg/dL; *P <* 0.001) and triglycerides (AO^Pos^: 132.8 ± 116.6; AO^Neg^: 80.8 ± 44.5 mg/dL; *P <* 0.001) (Table 2). The prevalence of liver disease and other inflammation was not different in AO^Neg^ and AO^Pos^ groups in both gender. However, AO^Pos^ group had higher prevalence of diabetes than AO^Neg^ group (men: AO^Pos^ v.s AO^Neg^ = 23.1% v.s 7.9%; *P <* 0.001; women AO^Pos^ v.s AO^Neg^ = 24.3% v.s 3.0%; *P <* 0.001) (Table 2).

**Table 2:** Distribution of biochemistry examination and chronic diseases in participants with abdominal obesity (AO^Pos^) and without (AO^Neg^). [Mean (SD) or Number (%)].

Characteristic	Men			Women		
	AO^Neg^	AO^Pos^	*P*	AO^Neg^	AO^Pos^	*P*
	(n = 847)	(n = 619)		(n = 666)	(n = 744)	
Cholesterol (mg/dL)	186.5 (40.2)	201.4 (41.7)	< 0.001	187.8 (40.5)	203.7 (44.0)	< 0.001
HDL cholesterol (mg/dL)	48.5 (13.7)	44.9 (13.2)	< 0.001	61.7 (15.8)	53.8 (13.6)	< 0.001
Triglycerides (mg/dL)	127.5 (123.7)	195.4 (255.8)	< 0.001	80.8 (44.5)	132.8 (116.6)	< 0.001
Fasting glucose (mg/dL)	96.5 (27.5)	110.0 (42.4)	< 0.001	90.0 (23.8)	110.0 (44.2)	< 0.001
Fasting insulin (mU/L)	8.0 (8.6)	12.5 (13.6)	< 0.001	6.3 (17.1)	11.3 (16.7)	< 0.001
HOMA-IR	1.99 (2.50)	3.63 (6.39)	< 0.001	1.38 (2.55)	3.26 (4.91)	< 0.001
Liver disease^a^ (%)	108 (12.8%)	87 (14.1%)	0.468	60 (9.0%)	77 (10.4%)	0.396
Renal disease (%)	28 (3.3%)	21 (3.4%)	0.927	6 (0.9%)	23 (3.1%)	0.004
Other inflammation^b^ (%)	18 (2.1%)	17 (2.8%)	0.442	44 (6.6%)	36 (4.8%)	0.152
Diabetes^c^ (%)	67 (7.9%)	143 (23.1%)	< 0.001	20 (3.0%)	181 (24.3%)	< 0.001

Abbreviations: AO = abdominally obese

*p*: indicates whether demographic characteristics and serum PCDD/Fs differ by AO status (Wilcoxon Rank-Sum test for continuous variables and χ^2^ test for categorical variables).

^a^ Self-report of having a Chronic hepatitis B/C infection, or hepatic steatosis or Liver Gallstones

^b^ Self-report of having foot or urinary tract infection

^c^ Self-report of (1) their fasting plasma glucose was ≥126 mg/dL, or (2) they reported a history of physician-diagnosed type 1 or

type 2 diabetes, or (3) they were currently using insulin and oral hypoglycemic agents.

In AO^Pos^ men, a Pearson correlation showed that WHR was significantly associated with Sys BP (r = 0.312) and Dia BP (r = 0.263) (both *P* < 0.001), and moderately-to-strongly associated with CHOL (r = 0.233; *P* < 0.001), Glucose (r = 0.216; *P <* 0.001), and TG (r = 0.212; *P <* 0.001). Moreover, serum ln-TEQ_DF-1998_ levels were also significantly associated with WHR (r = 0.295; *P* < 0.001), Sys BP (r = 0.301; *P* < 0.001), Glucose (r = 0.220; *P* < 0.001), and HOMA-IR (r = 0.105; *P* < 0.001) (S1 Table).

In AO^Pos^ women, a Pearson correlation showed that WHR was significantly associated with Sys BP (r = 0.444) and Dia BP (r = 0.319) (both *P* < 0.001) and moderately-to-strongly associated with Glucose (r = 0.287; *P* < 0.001), TG (r = 0.291; *P <* 0.001), and CHOL (r = 0.174; *P <* 0.001) (S2 Table).

Moreover, serum ln-TEQ_DF-1998_ levels were also significantly associated with WHR (r = 0.374; *P* < 0.001), Sys BP (r = 0.476; *P* < 0.001), Dia BP (r = 0.248; *P* < 0.001), Glucose (r = 0.238; *P* < 0.001), and HOMA-IR (r = 0.147; *P* < 0.001).

We also explored the association between abdominal obesity and quintiles of each of the 17 congener levels in multiple logistic regression models (Tables 3 and 4).

**Table 3:** Adjusted ORs (95% CIs) of the prevalence of abdominal obesity by quintiles of the concentrations of the selected congeners (Men).

Congener	Quintile 1	Quintile 2	Quintile 3	Quintile 4	Quintile 5	*P*_*trend*_
2,3,7,8-TCDF	1	1.2 (0.83–1.8)	**2.0 (1.4**–**2.9)**	**2.4 (1.6**–**3.5)**	**3.0 (2.0**–**4.5)**	**< 0.001**
1,2,3,7,8-PeCDF	1	**1.6 (1.1**–**2.4)**	**2.3 (1.6**–**3.4)**	**3.0 (2.0**–**4.5)**	**4.2 (2.7**–**6.4)**	**< 0.001**
2,3,4,7,8-PeCDF	1	1.3 (0.86–1.9)	**2.0 (1.3**–**3.0)**	**2.7 (1.7**–**4.2)**	**2.9 (1.9**–**4.7)**	**< 0.001**
1,2,3,4,7,8-HxCDF	1	**1.8 (1.2**–**2.6)**	**1.9 (1.3**–**2.8)**	**2.6 (1.7**–**3.8)**	**2.4 (1.6**–**3.6)**	**< 0.001**
1,2,3,6,7,8-HxCDF	1	1.4 (0.94–2.1)	**2.1 (1.4**–**3.1)**	**2.4 (1.6**–**3.5)**	**2.1 (1.4**–**3.2)**	**< 0.001**
2,3,4,6,7,8-HxCDF	1	1.2 (0.83–1.8)	**1.6 (1.1**–**2.4)**	**1.6 (1.1**–**2.3)**	**1.9 (1.3**–**2.9)**	**< 0.001**
1,2,3,7,8,9-HxCDF	1	1.4 (0.94–2.0)	1.4 (0.98–2.1)	1.1 (0.78–1.7)	1.1 (0.75–1.6)	0.416
1,2,3,4,6,7,8-HpCDF	1	1.3 (0.91–1.9)	1.3 (0.87–1.8)	**1.6 (1.1**–**2.3)**	1.4 (0.96–2.0)	0.788
1,2,3,4,7,8,9-HpCDF	1	1.0 (0.66–1.4)	1.1 (0.78–1.6)	0.75 (0.52–1.1)	0.97 (0.67–1.4)	>0.950
OCDF	1	1.0 (0.72–1.5)	1.1 (0.79–1.6)	1.1 (0.76–1.6)	0.89 (0.62–1.3)	>0.950
2,3,7,8-TCDD	1	**1.6 (1.1**–**2.3)**	**2.1 (1.4**–**3.1)**	**2.1 (1.4**–**3.2)**	**3.2 (2.1**–**5.0)**	**< 0.001**
1,2,3,7,8-PeCDD	1	**1.8 (1.2**–**2.8)**	**2.6 (1.7**–**4.0)**	**2.7 (1.7**–**4.1)**	**3.6 (2.3**–**5.7)**	**< 0.001**
1,2,3,4,7,8-HxCDD	1	1.1 (0.77–1.7)	**1.5 (1.0**–**2.2)**	**1.7 (1.2**–**2.6)**	**2.2 (1.5**–**3.4)**	**< 0.001**
1,2,3,6,7,8-HxCDD	1	1.3 (0.9–1.9)	1.3 (0.87–1.8)	**1.6 (1.1–2.3)**	1.4 (0.96–2.0)	0.788
1,2,3,7,8,9-HxCDD	1	1.3 (0.87–1.9)	**1.9 (1.3**–**2.8)**	**2.2 (1.5**–**3.3)**	**2.6 (1.7**–**3.9)**	**< 0.001**
1,2,3,4,6,7,8-HpCDD	1	1.3 (0.92–2.0)	**1.6 (1.1**–**2.4)**	**2.1 (1.5**–**3.1)**	**2.4 (1.6**–**3.6)**	**< 0.001**
OCDD	1	1.2 (0.85–1.8)	1.4 (0.94–1.9)	1.2 (0.86–1.8)	1.3 (0.93–1.9)	0.608
Total PCDFs	1	**1.7 (1.2**–**2.5)**	**1.6 (1.1**–**2.4)**	**2.1 (1.4**–**3.1)**	**2.0 (1.3**–**3.0)**	**< 0.001**
Total PCDDs	1	1.1 (0.75–1.6)	1.3 (0.92–1.9)	1.3 (0.87–1.8)	1.4 (0.94–2.0)	0.062
Total PCDD/Fs	1	1.1 (0.76–1.6)	1.2 (0.85–1.8)	1.3 (0.88–1.8)	**1.4 (1.0–2.1)**	**0.019**

CI, confidence interval; OR, odds ratio; PCDD, polychlorinated dibenzofurans; PCDD, polychlorinated dibenzodioxins.

Adjusted for age, smoking habit, drinking, physical activity, cholesterol, and triglycerides.

**Table 4:** Adjusted ORs (95% CIs) of the prevalence of abdominal obesity by quintiles of the concentrations of the selected congeners (Women).

Congener	Quintile 1	Quintile 2	Quintile 3	Quintile 4	Quintile 5	*P*_*trend*_
2,3,7,8-TCDF	1	0.76 (0.53–1.1)	0.86 (0.57–1.3)	1.1 (0.72–1.7)	1.3 (0.82–2.1)	**< 0.001**
1,2,3,7,8-PeCDF	1	0.78 (0.54–1.1)	0.86 (0.57–1.3)	1.1 (0.69–1.7)	1.1 (0.65–1.7)	**< 0.001**
2,3,4,7,8-PeCDF	1	0.88 (0.60–1.3)	0.9 (0.6–1.3)	1.2 (0.78–1.8)	1.2 (0.73–1.9)	**< 0.001**
1,2,3,4,7,8-HxCDF	1	0.84 (0.57–1.2)	0.92 (0.61–1.4)	1.1 (0.69–1.7)	0.97 (0.61–1.6)	**< 0.001**
1,2,3,6,7,8-HxCDF	1	1.4 (0.95–2.0)	1.3 (0.88–1.9)	1.5 (0.97–2.2)	**2.0 (1.3**–**3.1)**	**< 0.001**
2,3,4,6,7,8-HxCDF	1	1.0 (0.65–1.4)	**1.4 (1.0**–**2.1)**	**1.6 (1.1**–**2.4)**	**1.7 (1.1**–**2.6)**	**0.008**
1,2,3,7,8,9-HxCDF	1	1.0 (0.66–1.4)	0.97 (0.66–1.4)	1.3 (0.90–2.0)	1.3 (0.87–2.0)	**0.014**
1,2,3,4,6,7,8-HpCDF	1	1.0 (0.71–1.5)	1.3 (0.88–1.9)	**1.6 (1.1**–**2.4)**	**1.9 (1.3–2.9)**	0.529
1,2,3,4,7,8,9-HpCDF	1	1.2 (0.84–1.8)	**1.7 (1.2–2.6)**	**2.2 (1.5–3.3)**	**3.0 (1.9–4.7)**	0.923
OCDF	1	0.71 (0.49–1.0)	0.72 (0.47–1.1)	0.9 (0.56–1.4)	1.2 (0.69–2.0)	>0.950
2,3,7,8-TCDD	1	1.0 (0.69–1.5)	0.94 (0.63–1.4)	1.2 (0.82–1.9)	1.3 (0.81–2.0)	**< 0.001**
1,2,3,7,8-PeCDD	1	0.78 (0.54–1.1)	0.74 (0.50–1.1)	1.2 (0.80–1.8)	1.3 (0.80–2.0)	**< 0.001**
1,2,3,4,7,8-HxCDD	1	1.0 (0.67–1.4)	1.4 (0.92–2.0)	1.2 (0.78–1.7)	1.4 (0.91–2.1)	**< 0.001**
1,2,3,6,7,8-HxCDD	1	0.87 (0.59–1.3)	1.2 (0.79–1.7)	**1.7 (1.1–2.5)**	1.3 (0.88–2.0)	**< 0.001**
1,2,3,7,8,9-HxCDD	1	0.95 (0.65–1.4)	0.88 (0.60–1.3)	0.96 (0.65–1.4)	1.1 (0.77–1.7)	**< 0.001**
1,2,3,4,6,7,8-HpCDD	1	1.3 (0.91–2.0)	1.0 (0.70–1.5)	1.2 (0.79–1.7)	**1.6 (1.1**–**2.3)**	**< 0.001**
OCDD	1	0.96 (0.66–1.4)	0.88 (0.60–1.3)	0.99 (0.68–1.5)	1.2 (0.81–1.8)	**< 0.001**
Total PCDFs	1	0.74 (0.50–1.1)	0.69 (0.46–1.0)	1.2 (0.81–1.8)	1.0 (0.66–1.56)	**< 0.001**
Total PCDDs	1	1.0 (0.71–1.5)	1.0 (0.70–1.5)	1.4 (0.94–2.1)	1.5 (0.98–2.3)	**< 0.001**
Total PCDD/Fs	1	1.1 (0.74–1.6)	1.1 (0.72–1.6)	1.3 (0.90–2.0)	1.6 (1.0–2.4)	**< 0.001**

CI, confidence interval; OR, odds ratio; PCDD, polychlorinated dibenzofurans; PCDD, polychlorinated dibenzodioxins.

Adjusted for age, sex, smoking habit, drinking, physical activity, cholesterol, and triglycerides.

**Fig 1:**
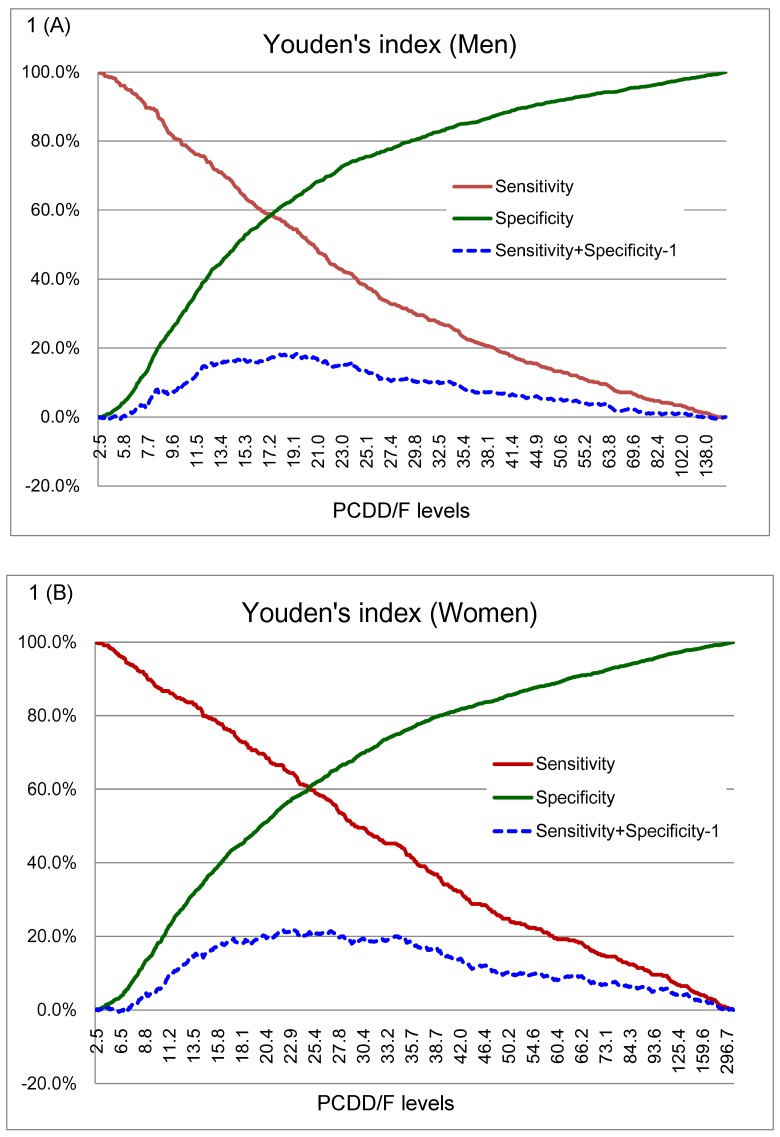
Two-graph receiver operator characteristic (ROC) curves for HOMA-IR against serum PCDD/F levels. These graphs indicate changes in sensitivity and specificity based on changes in the serum PCDD/F levels for (A) men and (B) women. Youden’s index (sensitivity + specificity − 1) is also shown. Abbreviations: HOMA-IR, homeostasis model assessment of insulin resistance.

The strength of associations between levels of selected congeners and the prevalence of abdominal obesity was correlated with toxic equivalency factors (TEFs) even after adjustment for confounding factors. Significant trends in association between male abdominal obesity and low-chlorinated congeners such as 2,3,7,8-TCDF; 1,2,3,7,8-PeCDF; 2,3,4,7,8-PeCDF; 2,3,7,8-TCDD; 1,2,3,7,8-PeCDD; and 1,2,3,7,8,9-HxCDD was found (*P*_trend_ < 0.001, and ORs for quintile 5 > 2.5) (Table 3). The highest quintiles of 1,2,3,7,8-PeCDF; 1,2,3,7,8-PeCDD; 2,3,7,8-TCDD; 2,3,7,8-TCDF; and 2,3,4,7,8-PeCDF had the top five AORs (95% CI) of 4.2 (2.7–6.4), 3.6 (2.3–5.7), 3.2 (2.1–5.0), 3.0 (2.0–4.5), and 2.9 (1.9–4.7), respectively (Table 3). In women, we found significant trends for associations with abdominal obesity for these low-chlorinated congeners: 1,2,3,6,7,8-HxCDF; 1,2,3,4,6,7,8-HpCDF; and 1,2,3,4,7,8,9-HpCDF (ORs for quintile 5 > 1.8) (Table 4). The highest quintiles of 1,2,3,4,7,8,9-HpCDF; 1,2,3,6,7,8-HxCDF; and 1,2,3,4,6,7,8-HpCDF had the top three AORs (95% CI) of 3.0 (1.9–4.7), 2.0 (1.3–3.1), and 1.9 (1.3–2.9), respectively (Table 4). Two graph ROC curves for insulin resistance against serum PCDD/Fs are shown in Fig 1. These graphs indicate changes in the sensitivity and specificity according to the cutoff value for serum PCDD/Fs. Youden’s index (sensitivity + specificity −1) is also shown in Fig 1. The cutoff values were 19.35 for men and 23.25 pg WHO_1998_-TEQ_DF_/g lipid for women. Sensitivity and specificity for the cutoff values were 55% and 64% for men and 64% and 58% for women, respectively. We found that men, but not women, with higher serum TEQ_DF-1998_ levels or who were AO^Pos^ had a significantly higher risk for abnormal insulin resistance even after adjustment for confounding factors, (*P*_trend_ < 0.001) (Tables 5 and 6). In men, the joint highest serum ln-TEQ_DF-1998_ levels and abdominal obesity was associated with elevated insulin resistance at 5.0 times the odds of the joint lowest (AOR: 5.23, 95% CI: 3.53–7.77).

**Table 5:** Association between serum TEQ_DF-1998_ and the risk of insulin resistance in participants (Men = 1466).

	Total	Insulin Resistance^b^	OR
Variables	(N = 1466)	n (%)	(95% CI)
Age (years)			
< 40	612 (41.8)	126 (20.6)	1
40–60	549 (37.5)	187 (34.1)	1.10 (0.80–1.51)
> 60	305 (20.7)	89 (29.2)	0.76 (0.50–1.14)
Smoking			
Never	634 (43.2)	162 (25.6)	1
Yes	832 (56.8)	240 (28.9)	0.88 (0.68–1.16)
Drinking			
No	1063 (72.5)	272 (25.6)	1
Yes	403 (27.5)	130 (32.3)	0.87 (0.65–1.16)
Physical activity^a^			
No	891 (61.5)	245 (27.5)	1
Yes	557 (38.5)	153 (27.5)	1.07 (0.82–1.39)
TG abnormality			
No	1015 (69.2)	199 (19.6)	1
Yes	451 (30.8)	203 (45.0)	1.08 (0.83–1.39)
CHOL abnormality			
No	861 (58.7)	207 (24.0)	1
Yes	605 (41.3)	195 (32.2)	2.88 (2.20–3.79)
Abdominal Obesity^†^/Dioxins^‡^			
AO^Neg^/Dioxins low	588 (40.1)	79 (13.4)	1
AO^Pos^/Dioxins low	277 (18.9)	104 (37.6)	**3.08 (2.14–4.43)**
AO^Neg^/Dioxins high	252 (17.2)	66 (26.2)	**2.46 (1.63–3.70)**
AO^Pos^/Dioxins high	349 (23.8)	153 (43.8)	**5.23 (3.53–7.77)**

Interaction term 1.20 (0.93–1.55)

^†^ Abdominal obesity: waist-to-hip ratio (WHR) was defined as > 0.9 in men.

^‡^ Low: Serum TEQ_DF-1998_< 19.35 pg WHO_98_-TEQ_DF_/g lipid; High: 19.35 ≤ serum TEQ_DF-1998_

Abbreviations: OR = odds ratio; CI = confidence interval.

^a^ Self-report of a brisk daily 30-minute walk or even a 15-minute run.

^b^ Homoeostasis model assessment of insulin resistance ≥ 75th percentile: ≥2.78.

**Table 6:** Association between serum TEQ_DF-1998_ and the risk of insulin resistance in participants (Women = 1410).

	Total	Insulin Resistance^b^	OR
Variables	(N = 1410)	n (%)	(95% CI)
Age (years)			
< 40	574 (40.7)	67 (11.7)	1
40–60	507 (36.0)	129 (25.4)	1.37 (0.91–2.04)
> 60	329 (23.3)	120 (36.5)	1.57 (0.95–2.58)
Smoking			
Never	1348 (95.6)	304 (22.6)	1
Yes	62 (4.4)	12 (19.4)	0.89 (0.43–1.86)
Drinking			
No	1375 (97.5)	313 (22.8)	1
Yes	35 (2.5)	3 (8.6)	0.41 (0.12–1.41)
Physical activity^a^			
No	949 (68.4)	191 (20.1)	1
Yes	439 (31.6)	115 (26.2)	1.10 (0.82–1.49)
TG abnormality			
No	1164 (82.6)	185 (15.9)	1
Yes	246 (17.4)	131 (53.3)	0.96 (0.72–1.29)
CHOL abnormality			
No	821 (58.2)	154 (18.8)	1
Yes	589 (41.8)	162 (27.5)	3.58 (2.59–4.94)
Abdominal Obesity^†^/Dioxins^‡^			
AO^Neg^/Dioxins low	462 (32.8)	31 (6.7)	1
AO^Pos^/Dioxins low	285 (20.2)	83 (29.1)	**4.22 (2.66–6.71)**
AO^Neg^/Dioxins high	191 (13.6)	18 (9.4)	1.11 (0.58–2.12)
AO^Pos^/Dioxins high	472 (33.5)	184 (39.0)	**4.57 (2.7.-7.64)**

^†^ Abdominal obesity: waist-to-hip ratio (WHR) was defined as > 0.8 in women.

^‡^ Low: Serum TEQ_DF-1998_ < 23.25 pg WHO_98_-TEQ_DF_/g lipid; High: 23.25 ≤ serum TEQ_DF-1998_

Abbreviations: OR = odds ratio; CI = confidence interval.

^a^ Self-report of a brisk daily 30-minute walk or even a 15-minute run.

^b^ Homoeostasis model assessment of insulin resistance ≥ 75th percentile: ≥2.78.

## Discussion

We found that TG, glucose, and serum PCDD/Fs were more strongly associated with the WHR than with waist circumference, BMI, or body fat; however, the differences in correlation were not significant. Obesity is associated with an increased release of free fatty acids (FFAs) and an abnormal secretion of adipokines [21–23], which can adversely affect how insulin acts; therefore, both FFAs and adipokines potentially link obesity with insulin resistance.

Visceral and subcutaneous fat make different contributions to insulin resistance. Visceral fat is more sensitive to insulin’s lipolytic effect than is subcutaneous fat [21]. Furthermore, FFAs released from visceral fat will go directly to the liver by portal circulation, whereas FFAs derived from subcutaneous fat are secreted into the systemic circulation [24, 25]. The increased flux of FFAs from visceral fat through the liver can promote gluconeogenesis and hepatic insulin resistance and lead to an accelerated synthesis of very-low-density lipoprotein and increased triglyceride levels [25, 26].

12 of the selected 17 congeners, widely distributed among PCDDs and PCDFs, had trends for association with abdominal adiposity. In men, the highest quintiles of 1,2,3,7,8-PeCDF; 1,2,3,7,8-PeCDD; 2,3,7,8-TCDD; 2,3,7,8-TCDF; and 2,3,4,7,8-PeCDF had the top five AORs (95% CI) of 4.2 (2.7–6.4), 3.6 (2.3–5.7), 3.2 (2.1–5.0), 3.0 (2.0–4.5), and 2.9 (1.9–4.7), respectively.

In women, the highest quintiles of 1,2,3,4,7,8,9-HpCDF; 1,2,3,6,7,8-HxCDF; and 1,2,3,4,6,7,8-HpCDF had the top three AORs (95% CI) of 3.0 (1.9–4.7), 2.0 (1.3–3.1), and 1.9 (1.3–2.9), respectively. We found associations of PCDD/Fs with the prevalence of abdominal adiposity. These associations in our TEQ-based analyses agreed with the results of our congener-specific analyses. We found associations of PCDD/Fs with the prevalence of abdominal obesity; in contrast to a background survey [27], who reported a narrow range of exposure levels and found no such associations. As we know, there are no other published studies discussed that high levels of exposure to dioxins are associated with abdominal obesity in a well-defined cohort.

Inflammation of the adipose tissue is one of the hallmarks of obesity, and the inflammatory phenotype is critical in metabolic diseases. POPs induce proinflammatory genes in rodent adipose cells [28]. Similar effects were found in human adipocytes [29]. A cross-sectional study [30] reported that POPs and metals exposure at levels below the median had varying effects on the body size of Flemish adolescents. It also said that BMI was negatively associated with exposure to HCB, p,p′-DDE, and di-ortho PCBs: 138, 153, & 180. There was a positive association between BMI and dioxin-like PCB 118 in boys and girls. Arsenescu et al. (2008) showed, in vivo and in vitro, that mice exposed to low-dose dioxin-like PCB 77 underwent proinflammatory adipokine expression and increased adipocyte differentiation, both of which are involved in promoting adiposity and body weight increases [8]. In Magueresse-Battistoni et al. (2013), mice were fed a high-fat diet, to which low doses of dioxin and PCB had been added throughout their lives [31]. Certain food contaminants are likely to cause metabolic disorders, or of worsening them, especially when they accompany a high-fat diet. Magueresse-Battistoni et al. found that the effects of these contaminants were highly gender-dependent. Females showed degenerated glucose intolerance and an altered estrogen pathway. Males showed cholesterol and lipid metabolism levels that changed for the worse.

We found that abdominal adiposity, measured using the WHR was a consistently strong predictor of glucose levels and, consequently, potential insulin resistance. POPs are insulin antagonists in cellular models of adipocytes. For example, dioxin inhibited the expression of glucose transporter Glut4 and lipoprotein lipase in 3T3-F442a cells [28]. This anti-insulin effect is not general and consistent for all genes, however. Indeed, whereas dioxin was an insulin antagonist in certain genes, like the IGFBP1 gene in hepatocytes [32], it had a different effect on other genes, like the liver PEPCK gene, because it tended to inhibit both insulin and glucose levels gluconeogenesis in this tissue [33]. We previously reported a significant increase in insulin resistance across the serum PCDD/F categories (*P*_trend_ < 0.001). After confounding factors had been adjusted for, a positive association was still found between serum dioxins and the prevalence of insulin resistance [17].

The study revealed that men were more affected by PCDD/Fs than were women because their obesity-induced glucose intolerances worsened. We have also shown that low doses of contaminants do in fact affect humans chronically exposed to PCDD/Fs, especially when the contaminants are combined with abdominal adiposity.

Abdominal obesity is the outstanding obesity-related predictor of type 2 DM [34]. In addition, lifetime's consumption of a high-fat diet that contains low doses of dioxins will exacerbate metabolic disorders. However, it is uncertain to what degree abdominal obesity can be used as an alternative measure for insulin resistance in people who are moderately exposed to environmental polluting dioxins. In this study, we still found that PCDD/Fs are associated with increased abdominal obesity, and that serum TEQ_DF-1998_ is an important determinant of abdominal obesity independent of age, sex, smoking, alcohol drinking, physical activity, cholesterol, and triglycerides. In this study, we use the following criteria to define diabetes, such as (1) fasting plasma glucose≥126 mg/dL, or (2) they reported a history of physician-diagnosed diabetes, or (3) currently using insulin and oral hypoglycemic agents. In fact, we should use the oral glucose tolerance test (OGTT), to diagnose diabetes, or prediabetes. According to the National Institutes of Health (NIH), the OGTT it is better able to diagnose high blood glucose after a glucose challenge than the fasting blood glucose test. Moreover, there are many surrogate indices using glucose and insulin levels suggested as alternative measures of insulin resistance. As we all know, the gold standard approach for measuring insulin resistance is euglycemic-hyperinsulinemic clamp [35]; but these are complicated and invasive procedures, and they are generally impractical for use outside. Therefore, for epidemiological studies, screenings of possible high-risk populations, simpler methods are needed. These surrogate indices consists of homeostasis model assessment of insulin resistance (HOMA-IR) [36], quantitative insulin sensitivity check index (QUICKI) [37], Matsuda index [38], and the new simple index assessing insulin sensitivity using oral glucose tolerance test (SI_is_OGTT) [39]. The HOMA-IR, and QUICKI methods have been the most frequently used techniques in clinical or field study. Because they require only a single venipuncture in the fasting state and do not need for intravenous access. Matsuda index and SI_is_OGTT are models that use dynamic glucose and insulin values obtained during oral glucose tolerance tests (OGTT). The OGTT is applicable for large-scale screening and for repeat studies with least risk because no intravenous access is needed. However, OGTTs are more difficult to perform than simple measurements of fasting glucose and insulin levels [40]. The HOMA-IR is closely correlated with the insulin sensitivity index assessed by euglycemic clamp in only a few patients with type 2 diabetes [36]. Several studies also reported that HOMA-IR can provide a good correlation in the clamp studies in a relatively greater number of diabetic subjects [41, 42]. However, the HOMA-IR also present relatively low value when the insulin secretion decreases in people with advanced type 2 diabetes, because the HOMA-IR is acquired by multiplying the fasting glucose and insulin levels. On the other hand, several scholars have recently provided methods to assess insulin sensitivity using an oral glucose tolerance test (OGTT), which can assess insulin sensitivity in nondiabetic subjects [43]. Even in type 2 diabetes, the Matsuda index was correlated to clamp-derived insulin sensitivity. Although these parameters from the OGTT decrease with worsening of glucose tolerance, the values inversely increase once the total insulin secretion declined.

There are some limitations in our study. First, the cross-sectional design prevents us from making any conclusive statement about the temporality of the observed associations. In addition, we have problem ruling out the presence of additional unknown risk factors that we cannot control in our analyses. It is the first large, population-based survey of abdominal obesity and insulin resistance in people exposed to moderate-to-high levels of dioxin. Our findings support our hypothesis that serum TEQ_DF-1998_ and abdominal obesity affect the association with insulin resistance in general populations and that serum PCDD/F levels, independent of overall adiposity, predicting increased insulin resistance.

## Supporting Information

S1 Table Pearson correlation coefficients among abdominal obesity components, and serum PCDD/F levels (Men). (DOCX)

S2 Table Pearson correlation coefficients among abdominal obesity components, and serum PCDD/F levels (Women). (DOCX)
