# Supplementary material for: Abdominal Obesity and Insulin Resistance in People Exposed to Moderate-to-High Levels of Dioxin
Source: PLoS One. 2016 Jan 11;11(1):e0145818. doi: 10.1371/journal.pone.0145818 (PMC4713838; doi:10.1371/journal.pone.0145818)
Supplement: S1 Table — (DOCX) [file pone.0145818.s001.docx]

**S1 Table.** Pearson correlation coefficients among abdominal obesity components, and serum PCDD/F levels (Men)

| **Variable** | Age | Waist | WHR | Body Fat | BMI | Sys BP | Dia BP | CHOL | HDL | TG | Glucose | IR |
| --- | --- | --- | --- | --- | --- | --- | --- | --- | --- | --- | --- | --- |
| Waist | 0.247^**^ | 1 |  |  |  |  |  |  |  |  |  |  |
| WHR | 0.397^**^ | 0.759^**^ | 1 |  |  |  |  |  |  |  |  |  |
| Body Fat | 0.459^**^ | 0.756^**^ | 0.587^**^ | 1 |  |  |  |  |  |  |  |  |
| BMI | 0.029 | 0.844^**^ | 0.521^**^ | 0.677^**^ | 1 |  |  |  |  |  |  |  |
| Sys BP | 0.478^**^ | 0.310^**^ | 0.312^**^ | 0.395^**^ | 0.217^**^ | 1 |  |  |  |  |  |  |
| Dia BP | 0.118^**^ | 0.332^**^ | 0.263^**^ | 0.302^**^ | 0.322^**^ | 0.573^**^ | 1 |  |  |  |  |  |
| CHOL | 0.216^**^ | 0.204^**^ | 0.233^**^ | 0.253^**^ | 0.159^**^ | 0.144^**^ | 0.161^**^ | 1 |  |  |  |  |
| HDL | 0.069^**^ | -0.234^**^ | -0.150^**^ | -0.180^**^ | -0.254^**^ | 0.010 | -0.058^*^ | 0.164^**^ | 1 |  |  |  |
| TG | 0.008 | 0.203^**^ | 0.212^**^ | 0.144^**^ | 0.165^**^ | 0.077^**^ | 0.142^**^ | 0.379^**^ | -0.299^**^ | 1 |  |  |
| Glucose | 0.247^**^ | 0.159^**^ | 0.216^**^ | 0.177^**^ | 0.080^**^ | 0.153^**^ | 0.094^**^ | 0.127^**^ | -0.044 | 0.120^**^ | 1 |  |
| IR | 0.069^**^ | 0.247^**^ | 0.167^**^ | 0.212^**^ | 0.231^**^ | 0.110^**^ | 0.120^**^ | 0.094^**^ | -0.128^**^ | 0.179^**^ | 0.409^**^ | 1 |
| PCDD/Fs^†^ | 0.583^**^ | 0.185^**^ | 0.295^**^ | 0.276^**^ | 0.063^*^ | 0.301^**^ | 0.093^**^ | 0.090^**^ | 0.078^**^ | -0.013 | 0.220^**^ | 0.105^**^ |

^†^ PCDD/F levels were log-transformed.

^*^ *P* < 0.05, ^**^*P* < 0.01, ^***^*P* < 0.001 (two-tailed test).

Abbreviations: WHR = the ratio of the circumference of the waist to that of the hips; Sys BP = systolic blood pressure; Dia BP = diastolic blood pressure; HDL = high density lipoprotein; TG = triglycerides; IR = HOMA IR (homeostatic model assessment insulin resistance).

**Supplemental Table 2** Pearson correlation coefficients among abdominal obesity components, and serum PCDD/F levels (Women)

| **Variable** | Age | Waist | WHR | Body Fat | BMI | Sys BP | Dia BP | CHOL | HDL | TG | Glucose | IR |
| --- | --- | --- | --- | --- | --- | --- | --- | --- | --- | --- | --- | --- |
| Waist | 0.453^**^ | 1 |  |  |  |  |  |  |  |  |  |  |
| WHR | 0.505^**^ | 0.753^**^ | 1 |  |  |  |  |  |  |  |  |  |
| Body Fat | 0.612^**^ | 0.783^**^ | 0.570^**^ | 1 |  |  |  |  |  |  |  |  |
| BMI | 0.245^**^ | 0.821^**^ | 0.438^**^ | 0.744^**^ | 1 |  |  |  |  |  |  |  |
| Sys BP | 0.671^**^ | 0.474^**^ | 0.444^**^ | 0.547^**^ | 0.335^**^ | 1 |  |  |  |  |  |  |
| Dia BP | 0.317^**^ | 0.403^**^ | 0.319^**^ | 0.406^**^ | 0.364^**^ | 0.589^**^ | 1 |  |  |  |  |  |
| CHOL | 0.315^**^ | 0.169^**^ | 0.174^**^ | 0.267^**^ | 0.131^**^ | 0.234^**^ | 0.190^**^ | 1 |  |  |  |  |
| HDL | -0.047 | -0.339^**^ | -0.273^**^ | -0.230^**^ | -0.305^**^ | -0.103^**^ | -0.104^**^ | 0.315^**^ | 1 |  |  |  |
| TG | 0.238^**^ | 0.325^**^ | 0.291^**^ | 0.293^**^ | 0.266^**^ | 0.234^**^ | 0.203^**^ | 0.300^**^ | -0.333^**^ | 1 |  |  |
| Glucose | 0.267^**^ | 0.302^**^ | 0.287^**^ | 0.290^**^ | 0.224^**^ | 0.293^**^ | 0.171^**^ | 0.272^**^ | -0.073^**^ | 0.311^**^ |  |  |
| IR | 0.216^**^ | 0.335^**^ | 0.256^**^ | 0.304^**^ | 0.308^**^ | 0.248^**^ | 0.151^**^ | 0.120^**^ | -0.179^**^ | 0.263^**^ | 0.379^**^ | 1 |
| PCDD/Fs^†^ | 0.722^**^ | 0.302^**^ | 0.374^**^ | 0.392^**^ | 0.154^**^ | 0.476^**^ | 0.248^**^ | 0.201^**^ | -0.044 | 0.208^**^ | 0.238^**^ | 0.147^**^ |

^†^ PCDD/F levels were log-transformed.

^*^ *P* < 0.05, ^**^*P* < 0.01, ^***^*P* < 0.001 (two-tailed test).

Abbreviations: WHR = the ratio of the circumference of the waist to that of the hips; Sys BP = systolic blood pressure; Dia BP = diastolic blood pressure; HDL = high density lipoprotein; TG = triglycerides; IR = HOMA IR (homeostatic model assessment insulin resistance).
