# Supplementary material for: Abdominal Obesity and Insulin Resistance in People Exposed to Moderate-to-High Levels of Dioxin
Source: PLoS One. 2016 Jan 11;11(1):e0145818. doi: 10.1371/journal.pone.0145818 (PMC4713838; doi:10.1371/journal.pone.0145818)
Supplement: S2 Table — (DOCX) [file pone.0145818.s002.docx]

**S2 Table.** Pearson correlation coefficients among abdominal obesity components, and serum PCDD/F levels (Women)

| **Variable** | Age | Waist | WHR | Body Fat | BMI | Sys BP | Dia BP | CHOL | HDL | TG | Glucose | IR |
| --- | --- | --- | --- | --- | --- | --- | --- | --- | --- | --- | --- | --- |
| Waist | 0.453^**^ | 1 |  |  |  |  |  |  |  |  |  |  |
| WHR | 0.505^**^ | 0.753^**^ | 1 |  |  |  |  |  |  |  |  |  |
| Body Fat | 0.612^**^ | 0.783^**^ | 0.570^**^ | 1 |  |  |  |  |  |  |  |  |
| BMI | 0.245^**^ | 0.821^**^ | 0.438^**^ | 0.744^**^ | 1 |  |  |  |  |  |  |  |
| Sys BP | 0.671^**^ | 0.474^**^ | 0.444^**^ | 0.547^**^ | 0.335^**^ | 1 |  |  |  |  |  |  |
| Dia BP | 0.317^**^ | 0.403^**^ | 0.319^**^ | 0.406^**^ | 0.364^**^ | 0.589^**^ | 1 |  |  |  |  |  |
| CHOL | 0.315^**^ | 0.169^**^ | 0.174^**^ | 0.267^**^ | 0.131^**^ | 0.234^**^ | 0.190^**^ | 1 |  |  |  |  |
| HDL | -0.047 | -0.339^**^ | -0.273^**^ | -0.230^**^ | -0.305^**^ | -0.103^**^ | -0.104^**^ | 0.315^**^ | 1 |  |  |  |
| TG | 0.238^**^ | 0.325^**^ | 0.291^**^ | 0.293^**^ | 0.266^**^ | 0.234^**^ | 0.203^**^ | 0.300^**^ | -0.333^**^ | 1 |  |  |
| Glucose | 0.267^**^ | 0.302^**^ | 0.287^**^ | 0.290^**^ | 0.224^**^ | 0.293^**^ | 0.171^**^ | 0.272^**^ | -0.073^**^ | 0.311^**^ |  |  |
| IR | 0.216^**^ | 0.335^**^ | 0.256^**^ | 0.304^**^ | 0.308^**^ | 0.248^**^ | 0.151^**^ | 0.120^**^ | -0.179^**^ | 0.263^**^ | 0.379^**^ | 1 |
| PCDD/Fs^†^ | 0.722^**^ | 0.302^**^ | 0.374^**^ | 0.392^**^ | 0.154^**^ | 0.476^**^ | 0.248^**^ | 0.201^**^ | -0.044 | 0.208^**^ | 0.238^**^ | 0.147^**^ |

^†^ PCDD/F levels were log-transformed.

^*^ *P* < 0.05, ^**^*P* < 0.01, ^***^*P* < 0.001 (two-tailed test).

Abbreviations: WHR = the ratio of the circumference of the waist to that of the hips; Sys BP = systolic blood pressure; Dia BP = diastolic blood pressure; HDL = high density lipoprotein; TG = triglycerides; IR = HOMA IR (homeostatic model assessment insulin resistance).
